# Socioeconomic and behavioural determinants of malaria among the migrants in gold mining, rubber and oil palm plantation areas in Myanmar

**DOI:** 10.1186/s40249-017-0355-6

**Published:** 2017-11-06

**Authors:** Htin Zaw Soe, Aung Thi, Ni Ni Aye

**Affiliations:** 1 University of Community Health, Magway, Myanmar; 2 Department of Public Health, Vector-borne Disease Control Program, nay Pyi Taw, Myanmar

**Keywords:** Malaria, Determinants, Migrant, Myanmar

## Abstract

**Background:**

Malaria is a major public health problem in Myanmar. Migrant populations are at high risk of contracting malaria and its control is more difficult than for settled population. Studies on malaria and migration are rare in Myanmar. This study was undertaken with the main objective of identifying socioeconomic and behavioural determinants of malaria among the migrant workers involved in gold mining, rubber and oil palm plantations.

**Methods:**

A cross-sectional analytic study was conducted using pretested interview-administered questionnaires among internal migrants (*n* = 406) in the malaria endemic townships of Shwegyin, Bago Region, Thanbyuzayat, Mon State and Kawthaung, Taninthayi Region from August to November, 2015. Data were collected by well-trained Basic Health Staff members in study areas, and then analysed by SPSS version 16.0 using Chi-square tests with significant level at 0.05.

**Results:**

Majority of participants were male, Bahmar nationals, married and with primary basic education level and below. The mean duration of migratory work was 4.51 years. 43.1% of them gave definite previous history of malaria within last two years during migration. 92.9% (377/406) of them always used bed nets. Malaria determinants found were male gender (OR = 1.84, 95% *CI*: 1.22–2.77; *P* = 0.0040), habit of going out at dawn (*OR* = 2.36, 95% *CI*: 1.58–3.52; *P* < 0.001), usual sleeping indoors (*OR* = 2.14, 95% *CI*: 1.04–4.42; *P* = 0.036), torn bed net or net with large hole(s) (*OR* = 2.0, 95% *CI*: 1.21–3.3; *P* = 0.006), habit of not always sleeping under a bed net at night (*OR* = 2.02, 95% *CI*: 1.15–3.52; *P* = 0.014), alcohol drinking (*OR* = 2.71, 95% *CI*: 1.73–4.26; *P* < 0.001) and failure to attend malaria health talk (*OR* = 1.78, 95% *CI*: 1.2–2.65; *P* = 0.004).

**Conclusions:**

The present study highlighted that it is warranted to launch an effective health education programme for malaria, and to encourage the proper use of insecticide-treated bed nets, blankets and/or mufflers and mosquito repellents to reduce the occurrence of malaria among the migrants.

## Background

International Organization for Migration (IOM) defines a migrant as any person who is moving or has moved across an international border or within a state away from his/her habitual place of residence, regardless of (i) the person’s legal status; (ii) whether the movement is voluntary or involuntary; (iii) what the causes for the movement are; or (iv) what the length of the stay is [1]. There are 214 million international migrants along with 740 million internal migrants over the world. One out of seven persons is on the move which is often cyclical and seasonal. The increasingly multi-directional massive movements of people with marked feminisation raises complex implications on global health throughout the phases of migration – before departure, during travel and transit, at destination and upon return. Population movement plays an important and complex role in the epidemiology of malaria. When travelling from low to high malaria transmission areas, they are more susceptible than the resident population. On the other hand migration from high to low transmission area will expose the previously malaria-free vectors to the infection. Migrants infected with malaria can serve as a reservoir and seed local outbreaks. This makes it difficult for countries that are linked by human mobility patterns to eliminate malaria. It can be shown that there are ‘*Plasmodium falciparum* migration communities’ around the world with much more infection-migration between the countries concerned than with the surrounding regions. Once drug resistance emerges, it can quickly spread along the human migration lines. It threatens progress towards malaria elimination and the control of artemisinin resistance. The 61st World Health Assembly Resolution on the Health of Migrants (WHA 61.17), adopted in May 2008, calls upon governments to promote migrant-sensitive health policies and to promote equitable access to health promotion and care for migrants [2, 3]. Malaria is also a major public health problem in Myanmar adding to people economic burden and reduced productivity. Out of 330 townships in the country, 284 are malaria endemic areas where 72% of Myanmar’s population resides. High risk groups include those dwelling near or in forest, plantation workers and migrants [4]. About 360,000 confirmed malaria cases and 300 deaths were reported annually on average in 2011–2014. In 2014 the cases fell to about 152,000 and deaths to 92 only [5]. In Myanmar migrants are usually working in gold mining, rubber plantation, dam construction, timber extraction and fishery. Magnitude of the problem in migrant populations was not known. If a malaria outbreak occurs in that population it is difficult to control the infection due to lack of relevant health facilities in their workplaces. The Myanmar Artemisinin Resistance Containment (MARC) Project was endorsed in 2011. One of seven objectives of that project is ‘to increase migrant/mobile populations’ access to and use of malaria diagnosis, treatment and vector control measures including personal protection [6, 7]. To achieve this objective a good understanding of the demographics, social determinants and practices of migrant workers is required to enable effective approaches and strategies to be applied and tailored to specific subsets of migrant workers. In social research considerable attempt was devoted to relate the risk of disease and sociocultural and behavioural factors [8]. The studies on malaria and migration had rarely been conducted in Myanmar. Thus this study was undertaken with a main purpose of identifying socioeconomic and behavioural determinants of malaria among the migrants involved in gold mining, rubber and oil palm plantation work in Myanmar.

## Methods

### Study design

It was a cross-sectional analytic study.

### Study areas

**Fig. 1:**
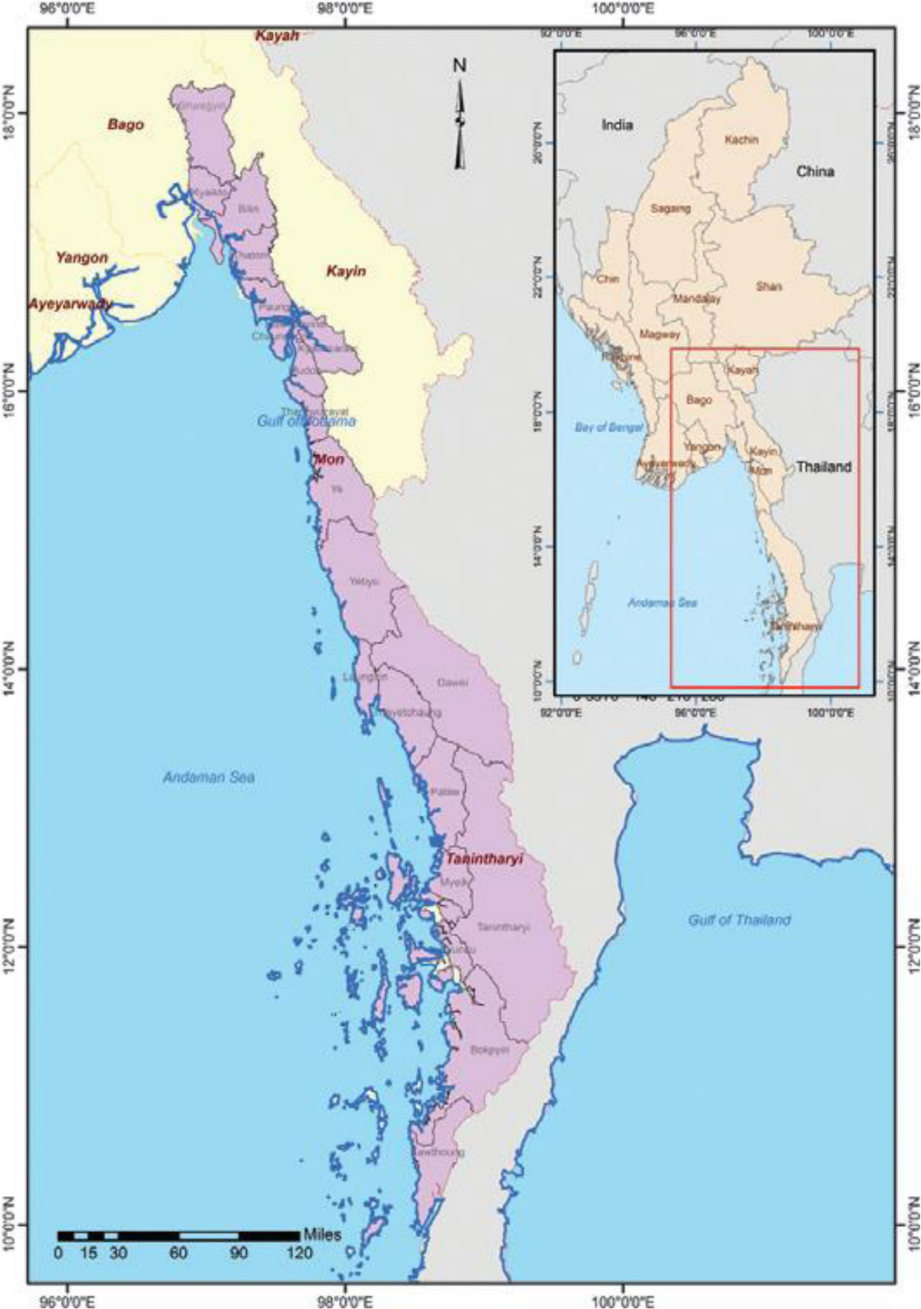
Map of study areas – Bago Region, Mon State and Tanintharyi Region *(Source: IOM, 2012; with a kind permission from IOM)*

The study areas were Shwegyin, Thanbyuzayat and Kawthaung townships. These areas were purposively selected because they are categorized as Tier I under the MARC Framework with evidence of malaria parasites with artemisinin resistance, widespread ecological and social risk factors for malaria transmission, and intensive population movement [9]. Shwegyin Township (c17° 57′ N, 96° 55′ E) is located in south-eastern part of Bago Region where most of migrants usually worked in gold mining places. Thanbyuzayat Township (c15° 57′ N, 97° 45′ E) is situated in southern part of Mon State where majority of migrants worked at rubber plantation sites. Kawthaung Township lies in southernmost part of Myanmar (c10° 2′ N, 98° 33′ E) in Tanintharyi Region where most of migrants were employed in oil palm plantation firms (Fig. 1.). Malaria morbidity rates in Shwegyin, Thanbyuzayat and Kawthaung Township were reported to be 46.5, 18.4 and 58.6 per 1000 populations, respectively in 2011 [9].

### Study population

Participants were temporary internal migrants and their family members who had left their residence of origin in various parts of Myanmar staying in study areas at least one year or over covering a malaria transmission season with or without their plans to return home upon completion of their specific work. They were aged 15 years and above and of both genders.

### Study period

It was from August to November, 2015.

### Sample size

As the proportions of malaria determinants among study populations were unknown the sample size was conventionally calculated as *n* = 1.96^2^ (0.5) (0.5) / 0.05^2^ = 384. To cover nonresponse 20% of the sample size (77) was added and final sample size became 461 (384 + 77). At least one third of 384 (ie. 128) was collected from each township.

### Sampling methods

A consecutive sampling method was used till a total required number of participants were obtained.

### Data collection methods and tools

Before data collection advocacy meetings were held with local administrative and health authorities in their offices in the study areas. The employers of the study populations in the areas were informed and their consents were verbally obtained. Next the study populations were mobilized at health centres at each township. Each day ten to twenty eligible migrants were invited to the health centres. They were explained about purpose and procedure of the study and their written informed consents were obtained. They were interviewed separately by well-trained Basic Health Staff (BHS) members from Regional/State vector-borne disease control teams concerned, using pre-tested interview-administered questionnaires to obtain socio-demographic and behavioural characteristics in relation to malaria occurrence (self-reported malaria in the last two years during migration).

### Data management

SPSS software version 16.0 was used. Data cleaning was done and incomplete or controversial questionnaire sets were discarded. Frequency tables were drawn and chi-squared tests were used to find associations between independent (determinants) and dependent variables (malaria occurrence). Odds ratios were calculated with 95% confidence interval to express strength of associations. Significant level was set at 0.05.

## Results

Out of 461 participants of the present study, 55 failed to give complete answers to all questions (ie. failure to mention important variable - age, duration of migration and history of malaria) in the questionnaire. Thus the remaining 406 were subjects of data analysis. Migrants were at mean age of 36 ± 12 years with males outnumbering females. Most of migrants were Bahmar nationals (90.4%), married individuals (70%), and with primary basic education level and below (59.3%) (Table 1). Their median monthly income was 131,000 Kyats. They moved from the residence of origin to places of destination (ie. workplaces) in three study areas of Shwegyin, Thanphyuzayat and Kawthaung. Most of the migrants (77.1%) in Shwegyin had moved from Bago Region, majority (41.5%) in Thanphyuzayat from Mon Region, and largest group (25.8%) in Kawthaung from Tanintharyi Region (Table 2). Migrants’ mean durations of migratory work in these three places of destination were 5.3, 4.96 and 3.58 years, respectively; and the overall mean duration was 4.51 years.

**Table 1:** Socio-demographic characteristics of migrants (*n* = 406)

Variable	Category	Frequency (%)
Ethnic group	Bahmar	367 (90.4)
	Mon	13 (3.2)
	Kayin	10 (2.5)
	Others	16 (3.9)
Marital status	Single	101 (24.9)
	Married	284 (70)
	Widowed	14 (3.4)
	Separated/divorced	7 (1.7)
Education	Illiterate	18 (4.4)
	3Rs	41 (10.1)
	Primary	182 (44.8)
	Middle	115 (28.3)
	High	38 (9.4)
	University	2 (0.5)
	Graduate	10 (2.5)

**Table 2:** Residence of origin by place of destination among the migrants (*n* = 406)

Residence of origin (Region/State)	Place of destination (township) (%)			Total
	Shewgyin	Thanphyuzayat	Kawthaung	
Yangon	1 (0.9)	4 (2.8)	8 (5)	13
Mandalay	3 (2.9)	0 (0)	37 (23.3)	40
Bago	81 (77.1)	35 (24.6)	27 (17)	143
Magway	2 (1.9)	0 (0)	2 (1.3)	4
Sagaing	0 (0)	0 (0)	1 (0.6)	1
Ayeyarwaddy	1 (0.9)	44 (31)	30 (18.9)	75
Tanintharyi	2 (1.9)	0 (0)	41 (25.8)	43
Mon	12 (11.4)	59 (41.5)	7 (4.4)	78
Kayin	0 (0)	0 (0)	3 (1.9)	3
Rakhine	3 (2.9)	0 (0)	3 (1.9)	6
Total	105 (100)	142 (100)	159 (100)	406

Socioeconomic and behavioural determinants of malaria among the migrants are also described in Table 3. Out of 406 participants, 175 (43.1%) gave definite history of suffering malaria within last two years. A definite history included symptoms of fever with chill and rigor for 3–7 days, seeking treatment from local BHS members and taking anti-malaria drugs and symptoms disappearing after treatment. Male gender (*OR* = 1.84, 95% *CI*: 1.22–2.77; *P* = 0.004), habit of going out at dawn (*OR* = 2.36, 95% *CI:* 1.58–3.52; *P* < 0.001), usual sleeping indoors (*OR* = 2.14, 95% *CI:* 1.04–4.42; *P* = 0.036), torn bed net or net with large hole(s) (*OR* = 2.0, 95% *CI*: 1.21–3.3; *P* = 0.006), habit of not always sleeping under a bed net at night (*OR* = 2.02, 95% *CI*: 1.15–3.52; *P* = 0.014), alcohol drinking (*OR* = 2.71, 95% *CI*: 1.73–4.26; *P* < 0.001), and failure to attend malaria health talk (*OR* = 1.78, 95% *CI:* 1.2–2.65; *P* = 0.004) were significantly associated with history of malaria occurrence. But usual bath place of migrants and habit of going out at dusk were not associated with it (*P* > 0.05). There were also highly significant associations between malaria morbidity and types of occupation (fire wood cutter and casual labourer) (*P* < 0.001) and the duration of migration (*P* < 0.001) (Tables 4 and 5).

**Table 3:** Socioeconomic and behavioural determinants of malaria among the migrants (*n* = 406)

Variable	Category	Malaria occurrence (%)			*OR* (95% *CI*)	*P*-value
		Present (*n*_1_ = 175)	Absent (*n*_2_ = 231)	Total (*n* = 406)		
Gender	Male	121(48.8)	127 (51.2)	248 (100)	1.84	0.004
	Female	54 (34.2)	104 (65.8)	158 (100)	(1.22–2.77)	
Usual bath place	At home	14 (53.8)	12 (46.2)	26 (100)	-	0.297
	In creek	71 (44.4)	89 (55.6)	160 (100)		
	At well	77 (39.3)	119 (60.7)	196 (100)		
	Others	13 (54.2)	11 (45.8)	24 (100)		
Habit of going out at dusk	Yes	97 (46.6)	111 (53.4)	208 (100)	1.34	0.141
	No	78 (39.4)	120 (60.6)	198 (100)	(0.91–1.99)	
Habit of going out at dawn	Yes	102 (54.3)	86 (45.7)	188 (100)	2.36	< 0.001
	No	73 (33.5)	145 (66.5)	218 (100)	(1.58–3.52)	
Usual sleeping place	Indoor	164 (44.8)	202 (55.2)	366 (100)	2.14	0.036
	Outdoor	11 (27.5)	29 (72.5)	40 (100)	(1.04–4.42)	
Torn bed net or net with large hole(s) (n = 377)	Yes	44 (55.7)	35 (44.3)	79 (100)	2.0	0.006
	No	115 (38.6)	183 (61.4)	298 (100)	(1.21–3.3)	
Not always sleeping under a bed net at night (*n* = 377)	Yes	33 (56.9)	25 (43.1)	58 (100)	2.02	0.014
	No	126 (39.5)	193 (60.5)	319 (100)	(1.15–3.52)	
Alcohol drinking	Yes	67 (60.9)	43 (39.1)	110 (100)	2.71	< 0.001
	No	108 (36.5)	188 (63.5)	296 (100)	(1.73–4.26)	
Ever attended malaria health talk	No	103 (50)	103 (50)	206 (100)	1.78	0.004
	Yes	72 (36)	128 (64)	200 (100)	(1.2–2.65)

**Table 4:** Type of occupation vs malaria occurrence (*n* = 406)

Type of occupation	Malaria occurrence (%)		Total (%)	*P*-value
	Present	Absent		
Rubber tree tapper	51 (32.5)	106 (67.5)	157 (100)	< 0.001
Casual labourer	42 (54.5)	35 (45.5)	77 (100)	
Oil palm plantation worker	18 (34.6)	34 (65.4)	52 (100)	
Communication worker	10 (43.1)	20 (56.9)	30 (100)	
Gold mine digger	11 (52.4)	10 (47.6)	21 (100)	
Fire wood cutter	16 (76.2)	5 (23.8)	21 (100)	
Others	27 (56.2)	21 (43.8)	48 (100)

**Table 5:** Duration of migration vs malaria occurrence (*n* = 406)

Duration of migration (year)	Malaria occurrence (%)		Total (%)	*OR* (95% *CI*)	*P*-value
	Present	Absent			
1–2	42 (31.8)	90 (68.2)	132 (100)	1	> 0.05
3–5	70 (41.9)	97 (58.1)	167 (100)	1.55 (0.96–2.5)	< 0.001
> 5	63 (58.9)	44 (41.1)	107 (100)	3.07 (1.81–5.2)	< 0.001 ^a^

^a^Overall significance

## Discussion

Migration is a process of moving, either across an international border (international migrants), or within a state (internal migrants). The structural inequalities experienced by many migrants have a significant impact on overall health and well-being. Migrant groups face different health challenges and have different levels of access to health and social services [10].

A total of 406 apparently healthy migrants from three study areas were investigated for socioeconomic and behavioural determinants of malaria. The respondents were not followed up over 4 months as different sites were visited and interviewed at different times over the study period. Most of them were young adults working at rubber plantation sites as rubber tree tappers to collect rubber milk, followed by casual labourers, oil palm plantation workers, communication workers, gold mine diggers and fire wood cutters. Other small groups were food vendors, dependent family members, a company manager, clerks, cooks, mechanics, a driver, etc. Among them 175 (43.1%) gave definite history of malaria that had occurred at least once in the last two years during migration. In a study at the Thai-Myanmar border area, about 40% of Thai and Karen migrants and almost 30% of Mon migrants suffered from malaria at least once [11].

In the present study male migrants were 1.8 times more likely to acquire malaria than females possibly due to failure to always sleep under a bed net among the former both at night and in daytime when they slept. In a study among local residents in malaria endemic area of Pyinmana Township, middle Myanmar (*n* = 154) male gender was also a clear risk factor for malaria (*OR* = 2.6, *P* = 0.004) [12]. It is also consistent with the finding in an Indonesian study where male had a higher risk of malaria [13]. In some societies, men have a greater occupational risk of catching malaria than women if they work in mines, fields or forests at peak biting times, or travel to areas of high endemicity for work [14]. Therefore male migrants should be a special attention in delivery of health education to take personal protective measures against mosquitoes.

Usual bath place of migrants was not a risk factor for malaria although creeks and built wells are usual breeding habitats of malaria vectors [15, 16]. Similarly habit of going out at dusk was not associated with malaria. It may be that individuals might have awareness of malaria vectors generally coming out at night and some kinds of personal protective measures like wearing long-sleeve garments. There were three types of rhythmic biting patterns of mosquitoes namely nocturnal (at night), diurnal (during the day time) and crepuscular (in twilight). Among these three patterns malaria vector *Anopheles* belongs to the nocturnal pattern [17].

Habit of going out at dawn was associated with malaria occurrence. Malaria vectors bite mainly between dusk and dawn [18]. It is possible that mosquitos may bite those going outside the human dwellings early in the morning when the environment is still dark. Migrants with this habit had a double chance of contracting malaria when compared with those who claimed not to go out at dawn. In the aforementioned study that habit was also found to be a risk factor for malaria (*OR* = 2.3, *P* = 0.01) [12]. So early morning out-goers should wear insecticide treated garments, and mufflers around their necks and use mosquito repellent to be applied over exposed body parts except face.

It was unexpectedly found that those who usually slept indoors had a double chance of acquiring malaria. Generally persons sleeping outdoors run a higher risk of being bitten by mosquitos. But in some areas mosquitoes are endophagic (ie. indoor biters). In the present study victims may most probably have been bitten by endophagic mosquitoes. Entomological surveys should be carried out to study bionomics of local mosquito vectors whether they are endophagic or exophagic. If they are endophagic, indoor residual spraying should be done in the migrant areas or migrants should sleep under bednets securely indoors.

In the present study, 92.9% (377/406) always used bed nets. In another study, 80.3% of migrants in rubber plantation (*n* = 105) and 76.2% of migrants in an oil palm plantation (*n* = 183) used bed nets [19]. Among 377 bed net using migrants in the present study, 79 (21%) said that their bed nets were torn or had large holes at sometimes. These migrants were twice as likely to suffer from malaria when compared with those possessing intact bed nets. Mosquitos may enter bed nets through torn sites or large holes any time. So bed nets torn or with large holes should be secured before migrants travel. Insecticide-treated bed nets (ITNs) or long lasting insecticide treated bed nets (LLINs) are strongly recommended to be used because even if the bed net is torn or has holes mosquitoes cannot enter bed net due to the repellent action of insecticide in ITN [20]. Some migrants cannot afford to buy ITN and in this case farm owners or employers should loan ITNs at cheap price to their migrants. Such loaning scheme was used in Cambodia [21]. According to the National Strategic Plan on Malaria LLINs will be provided to employers and then to their workers at construction and plantation sites and then later employers have to distribute LLINs at their own cost [22].

Another finding was association of alcohol drinking usually at night and malaria. Migrants with this habit had a 2.7 times higher risk of malaria because they may go to bed carelessly and sometimes unconsciously once they had finished drinking. So they may be bitten by mosquitos any time. To avoid malaria drinkers should take alcohols cautiously and go to bed carefully with their bed net rims tightly secured under mats or mattress that they sleep on. Policies for reduced alcohol use seem also of importance. A study has reported that the percent of mosquitos landing on volunteers was significantly higher after beer ingestion compared to before ingestion, showing that drinking alcohol stimulates mosquito attraction [23]. This finding has recently been confirmed by another group [24].

Migrants who never attended malaria health talk were 1.78 times more likely to get malaria probably because they may lack knowledge about prevention and control of malaria. Therefore health education talk should be undertaken widely in the migrant areas and migrants should be encouraged to attend.

Type of occupation and malaria morbidity was highly associated. Fire wood cutters had more chance of getting malaria and rubber tree tappers who usually worked at night had the lowest risk of malaria. This finding might disagree with a study from Indonesia where individuals with a workplace location in or near the forest requiring overnight stay with more exposure to malaria vectors had a higher risk of malaria [13].

Duration of migration was also highly linked to malaria morbidity. In this case, the longer the duration, the more the opportunity of contracting malaria among the migrants as the result of more travelling and exposure to mosquito bites.

Limitations of the present study are the use of a non-probability sampling method not fully representing to the study areas, and diagnosis of malaria by history taking only. Being a cross-sectional analytic study design it can only identify association but not prove causality.

## Conclusions

The present study highlighted that it is warranted to launch an effective health education programme of malaria, and to encourage proper use of insecticide-treated bed nets, blankets and/or mufflers and mosquito repellents to reduce the occurrence of malaria among the migrants.

## Abbreviations

3Rs: Reading writing and arithmetic in education
BHS: Basic health staff
IOM: International Organization for Migration
ITN: Insecticide treated bednet
LLIN: Long lasting insecticide treated bednet
MARC: Myanmar Artimesinin Resistance Containment
SPSS: Statistical package for social science
VBDC: Vector-borne Disease Control
WHA: World Health Assembly
